# Non-Invasive Delivery of CRISPR/Cas9 Ribonucleoproteins (Cas9 RNPs) into Cells via Nanoparticles for Membrane Transport

**DOI:** 10.3390/pharmaceutics17020201

**Published:** 2025-02-06

**Authors:** Toshihiko Tashima

**Affiliations:** Tashima Laboratories of Arts and Sciences, 1239-5 Toriyama-cho, Kohoku-ku, Yokohama 222-0035, Japan; tashima_lab@yahoo.co.jp

**Keywords:** CRISPR/Cas9, drug delivery system, nanodelivery, transmembrane substance delivery, receptor-mediated endocytosis/transcytosis

## Abstract

The clustered regularly interspaced short palindromic repeats/CRISPR-associated protein 9 (CRISPR/Cas9) system is a promising biotechnology tool for genome editing. However, in living organisms, several pharmacokinetic challenges arise, including off-target side effects due to incorrect distribution, low bioavailability caused by membrane impermeability, and instability resulting from enzymatic degradation. Therefore, innovative delivery strategies must be developed to address these issues. Modified nanoparticles offer a potential solution for the non-invasive delivery of CRISPR/Cas9 ribonucleoproteins (Cas9 RNPs). Cas9 RNPs encapsulated in nanoparticles are protected from enzymatic degradation, similar to how microRNAs are shielded within exosomes. It is well-established that certain materials, including proteins, are expressed selectively in specific cell types. For example, the α-7 nicotinic receptor is expressed in endothelial and neuronal cells, while the αvβ3 integrin is expressed in cancer cells. These endogenous materials can facilitate receptor-mediated endocytosis or transcytosis. Nanoparticles encapsulating Cas9 RNPs and coated with ligands targeting such receptors may be internalized through receptor-mediated mechanisms. Once internalized, Cas9 RNPs could perform the desired gene editing in the nucleus after escaping the endosome through mechanisms such as the proton sponge effect or membrane fusion. In this review, I discuss the potential and advantages of delivering Cas9 RNP-encapsulated nanoparticles coated with ligands through receptor-mediated endocytosis or transcytosis.

## 1. Introduction

**Figure 1:**
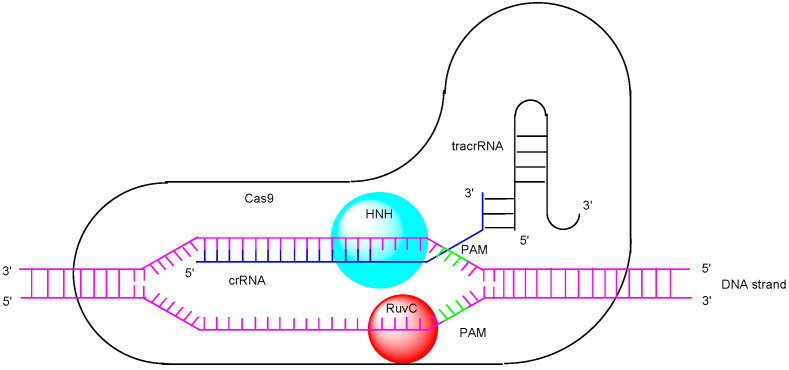
The interference mechanism of the type II CRISPR-Cas system involves the cleavage of target double-stranded DNA, originating from bacteria, at two endonuclease domains on the Cas9 protein: the HNH domain (named for its characteristic histidine and asparagine residues) and the RuvC domain (named after an *Escherichia coli* protein involved in DNA repair). CRISPR RNA (crRNA) and trans-activating CRISPR RNA (tracrRNA) recognize the target gene based on a specific protospacer adjacent motif (PAM).

**Figure 2:**
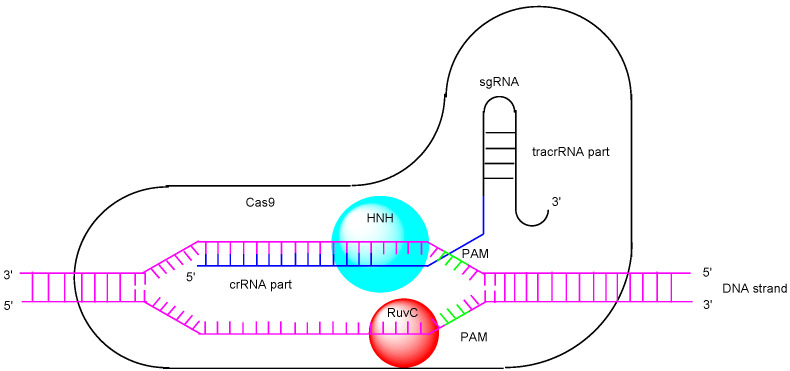
Single-guide RNA (sgRNA) is an engineered RNA molecule formed by combining CRISPR RNA (crRNA) and trans-activating CRISPR RNA (tracrRNA). PAM refers to the protospacer adjacent motif. The HNH domain is an endonuclease domain named for its characteristic histidine and asparagine residues, while the RuvC domain is an endonuclease domain named after a protein in *Escherichia coli* that plays a role in DNA repair.

Biotechnology has significantly contributed to the advancement of medicine, but the creation of genetically modified animals required for in vivo testing often presents challenges, such as the lack of desired embryonic stem cells. Therefore, efficient methods for obtaining knockout animals are needed. Currently, the clustered regularly interspaced short palindromic repeats (CRISPR)/CRISPR-associated (Cas) system is a revolutionary and simple tool for genome editing [1,2], alongside zinc finger nucleases (ZFNs) [3] and transcription activator-like effector nucleases (TALENs) [4]. In recognition of their pioneering work on this genome editing system, the 2020 Nobel Prize in Chemistry was awarded to Dr. Emmanuelle Charpentier and Dr. Jennifer Doudna. Although various Cas protein families have been discovered, Cas9 is a CRISPR-associated endonuclease that facilitates RNA-guided, site-specific DNA cleavage. In its natural form from *Streptococcus pyogenes*, Cas9 functions in conjunction with a dual-guide RNA, which consists of CRISPR RNA (crRNA) and trans-activating crRNA (tracrRNA). The mechanism of the type II CRISPR-Cas system can be summarized in three steps [5]: (i) Adaptation Stage: during the first infection, bacteria incorporate a new genetic spacer, derived from invading bacteriophages (phages), into the CRISPR array. (ii) crRNA Biogenesis: Upon reinfection, precursor CRISPR RNAs (pre-crRNAs) are transcribed from the CRISPR array. These pre-crRNAs are then processed into mature crRNAs with the help of tracrRNA and Cas9, facilitated by RNase III. (iii) Interference Stage (Figure 1): A complex of Cas9, tracrRNA, and crRNA binds to a short protospacer adjacent motif (PAM) sequence (3–6 nucleotides) located next to the target DNA protospacer sequence, enabling site-specific DNA targeting. Double-strand DNA is unwound around the PAM sequence. The single-stranded complementary DNA, which forms base pairs with the spacer of crRNA, is cleaved by the HNH domain (an endonuclease domain named for its characteristic histidine and asparagine residues), while the single-stranded non-complementary DNA is cleaved by the RuvC domain (an endonuclease domain named after an *Escherichia coli* protein involved in DNA repair). Ultimately, the targeted double-strand DNA break occurs in this manner [6]. Genome editing is achieved through non-homologous end joining (NHEJ) and homology-directed repair (HDR) following double-strand DNA cleavage. NHEJ is a repair mechanism that directly joins the ends of cleaved double-strand DNA. Occasionally, this process results in the insertion of unnecessary sequences, leading to repair errors. In bacteria, an excess of Cas9 can cause repeated double-strand DNA breaks, ultimately leading to repair errors [7]. On the other hand, HDR is a repair mechanism that uses a complementary sequence to guide precise DNA repair, allowing for accurate genome editing [8]. To improve efficiency, single-guide RNA (sgRNA) is artificially formed by connecting crRNA and tracrRNA (Figure 2). It is believed that CRISPR/Cas9 ribonucleoprotein (Cas9 RNP) is essential for the execution of gene editing in advance, providing an advantage over drug delivery and functional expression. However, the delivery of Cas9 into cells across the plasma membrane poses a challenge due to its large protein size. Nevertheless, in vitro or ex vivo gene editing can be performed through methods such as injection into fertilized eggs, chimeric antigen receptor (CAR)-T cells, or mesenchymal stem cells (MSCs) using techniques like lipofection (cationic lipid-mediated transfection), electroporation (electric pulse method), viral vector-based gene delivery, or microinjection [9]. However, in vivo gene editing in a non-invasive manner, without off-target side effects, presents several challenges that differ from in vitro gene editing using methods such as lipofection, electroporation, or microinjection. RNAs are degraded by RNases, while proteins are degraded by proteases. Interestingly, microRNAs (miRNAs) in exosomes released from donor cells into the systemic circulation are protected from such enzymatic degradation. A nanodelivery system using nanoparticles could potentially protect Cas9 RNP from enzymatic degradation. Furthermore, Cas9 RNP alone lacks the ability for cell internalization and cell selectivity. Currently, selective drug delivery systems, particularly those utilizing biological machinery such as receptor-mediated endocytosis/transcytosis, have been developed [10,11]. Therefore, in this perspective review, I explore the potential and implementation of selective, effective Cas9 RNP delivery into cells using nanoparticles to achieve non-invasive in vivo gene editing.

## 2. Discussion

### 2.1. Membrane Impermeability/Permeability of Substances

In drug discovery and development, membrane impermeability poses a significant pharmacokinetic challenge in living organisms. This is particularly evident for hydrophobic low-molecular-weight compounds and hydrophilic high-molecular-weight compounds, which cannot cross the blood–brain barrier (BBB) to enter the brain [12]. The BBB’s function is primarily attributed to physical barriers, including (i) the hydrophobic lipid bilayers of the capillary endothelial cell plasma membranes, (ii) the tight junctions between capillary endothelial cells, and (iii) periendothelial accessory structures supported by pericytes and astrocytes. Additionally, biological barriers play a role, such as (iv) excretion by efflux transporters like multidrug resistance protein 1 (MDR1, P-glycoprotein), which capture hydrophobic low-molecular-weight compounds that pass through the apical membrane of capillary endothelial cells. Consequently, the BBB significantly hinders the development of drugs for central nervous system (CNS) diseases. Moreover, cancer drug resistance is often attributed to the overexpression of MDR1 proteins at the apical membrane [13], posing a significant challenge to the development of effective cancer therapies. Therefore, it is crucial to establish selective and efficient transmembrane drug delivery systems by leveraging existing biological machinery, as guided by the principles of structuralism proposed by Dr. Claude Lévi-Strauss [14,15]. For example, nutrients such as glucose and amino acids are transported from systemic circulation into the brain across the BBB through solute carrier (SLC) transporters, including glucose transporter 1 (GLUT1) and the large neutral amino acid transporter (LAT). The mechanisms by which substances enter tissues, such as the brain, from the bloodstream across the endothelium or epithelium can be categorized into the following pathways: the paracellular pathway, including (i) passive diffusion through tight junctions, and the transcellular pathway, including (ii) passive diffusion through the lipid bilayers, (iii) carrier-mediated transport via solute carrier (SLC) transporters, (iv) receptor-mediated endocytosis/transcytosis involving specific receptors, (v) bystander macropinocytosis, and (vi) other less understood or unconventional transport methods [10]. The specific internalization mechanisms that occur depend on the properties of the substances, such as their molecular size, hydrophobicity or hydrophilicity, the structural environment in which they are situated, and their administration site. In some cases, multiple mechanisms occur simultaneously. Generally, substances are categorized based on molecular weight into low-molecular-weight compounds (molecular weight (MW) < approximately 500), high-molecular-weight compounds (MW > approximately 3000), and middle-molecular-weight compounds (MW approximately 500–3000). High-molecular-weight monoclonal antibodies, such as IgG molecules (approximately 14.2 nm in diameter and 150 kDa) [16], cannot pass through the pores of SLC transporters due to their size. Additionally, their hydrophilic nature prevents them from penetrating the membrane via passive diffusion. Nanoparticles (10–100 nm in diameter) face greater size limitations than monoclonal antibodies. It is well-known that nanoparticles coated with vectors such as anti-receptor monoclonal antibodies, cell-penetrating peptides (CPPs), or tumor-homing peptides (THPs) can cross cell membranes via receptor-mediated endocytosis. Among the various types of endocytosis, (i) clathrin-dependent endocytosis (with endosomal diameters of 85–150 nm) is the most prevalent. Other types include (ii) caveolae-dependent endocytosis (endosomal diameters of 50–100 nm), (iii) clathrin- and caveolae-independent endocytosis (endosomal diameters of approximately 90 nm), (iv) macropinocytosis (endosomal diameters of 0.2–5 µm), and (v) other less understood or mechanically complex forms of endocytosis [10]. Thus, Cas9 RNP-encapsulated nanoparticles can effectively cross membranes through carefully designed strategies that leverage structuralism to control biological machinery systems. The use of nanoparticles not only protects Cas9 RNP from degradative enzymes but also allows for easy surface modifications, enabling additional functionalities such as enhanced membrane internalization, cell selectivity, and immune tolerance. Indeed, nanoparticles encapsulating Cas9 RNP have been developed following these principles, as detailed below.

### 2.2. Nanoparticles Encapsulating Cas9 RNPs

#### 2.2.1. Distribution to Tissues, Such as the Liver, Involved in the Reticuloendothelial System

**Figure 3:**
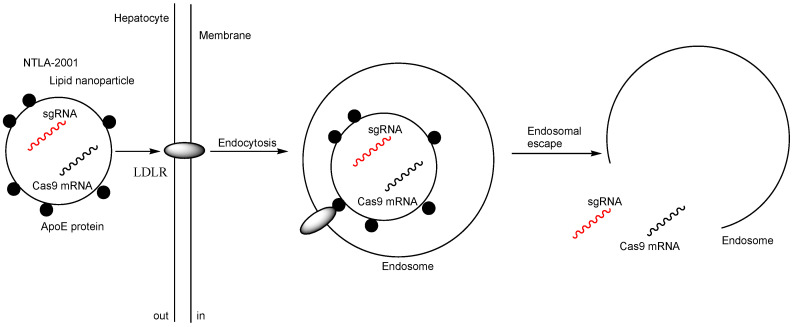
The pathway of NTLA-2001 involves lipid nanoparticles encapsulating Cas9 messenger RNA (mRNA) and single-guide RNA (sgRNA) targeting transthyretin (TTR), coated with apolipoprotein E (APOE) protein. APOE binds to low-density lipoprotein receptor (LDLR) to facilitate endocytosis. Endosomal escape is likely achieved through the breakdown of the lipid nanoparticles and the disruption of the endosomal membrane.

**Figure 4:**
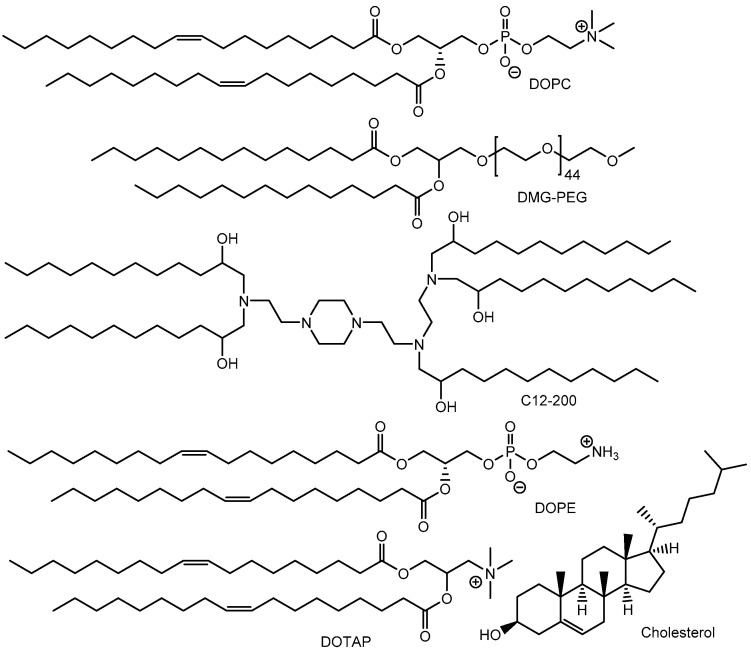
The structures of lipid nanoparticle components include 2,3-dioleoyl-glycero-1-phosphocholine (DOPC), 1,2-dimyristoyl-rac-glycero-3-methoxypolyethyleneglycol-2000 (DMG-PEG), cholesterol, 1,10-[[2-[4-[2-[2-[bis(2-hydroxydodecyl)amino]ethylamino]ethyl]piperazin-1-yl]ethyl]azanediyl]bis(dodecan-2-ol) (C12-200), 1,2-dioleoyl-sn-glycero-3-phosphoethanolamine (DOPE), and 1,2-dioleoyl-3-trimethylammonium-propane (DOTAP).

The establishment of in vivo genome editing has the potential to significantly expand therapeutic options for patients. Notably, (i) NTLA-2001, an intravenously administered lipid nanoparticle formulation encapsulating Cas9 messenger RNA (mRNA) and sgRNA targeting transthyretin (*TTR*), has been studied in a phase 1 clinical trial since 2020 for hereditary TTR amyloidosis with polyneuropathy (ATTRv-PN) and TTR amyloidosis-related cardiomyopathy (ATTR-CM) (NCT04601051). Preclinical studies demonstrated that, by day 28, the mean reduction in serum TTR protein concentration was 52% in the group receiving a dose of 0.1 mg per kilogram and 87% in the group receiving a dose of 0.3 mg per kilogram in humans [17]. TTR is a plasma transport protein responsible for carrying retinol (vitamin A) and thyroxine, primarily synthesized in the liver and the choroid plexus [18]. TTR amyloidosis is a gradually progressive condition resulting from the accumulation of misfolded TTR protein in tissues. Preclinical studies also revealed that NTLA-2001 complexes with apolipoprotein E (APOE) entered hepatocytes via receptor-mediated endocytosis utilizing low-density lipoprotein receptor (LDLR), leading to a reduction in serum TTR protein expression due to TTR gene knockout (Figure 3). It is believed that captured APOE acts as a vector facilitating internalization into hepatocytes. Endosomal escape is thought to occur through the breakdown of the lipid nanoparticle (LNP) and the disruption of the endosomal membrane. NTLA-2001 is formulated with proprietary ionizable lipids, combined with 2,3-dioleoyl-glycero-1-phosphocholine (DOPC), 1,2-dimyristoyl-rac-glycero-3-methoxypolyethylene glycol-2000 (DMG-PEG), and cholesterol (Figure 4) [19]. DOPC is a unique zwitterionic lipid that adopts a disordered superstructure characteristic of a fluid membrane [20]. Fundamentally, the delivery methods for Cas9 mRNA/sgRNA are the same as those for Cas9 RNPs, as both are encapsulated in nanoparticles. In the case of lipid nanoparticles, endosomal escape occurs through membrane fusion, membrane disruption, or the proton sponge effect. However, after being released from endosomes, Cas9 RNPs can immediately perform genome editing. In contrast, Cas9 mRNA must first undergo translation to produce the Cas9 protein. Table 1 presents studies that utilize either RNP delivery or mRNA/sgRNA delivery.

**Figure 5:**
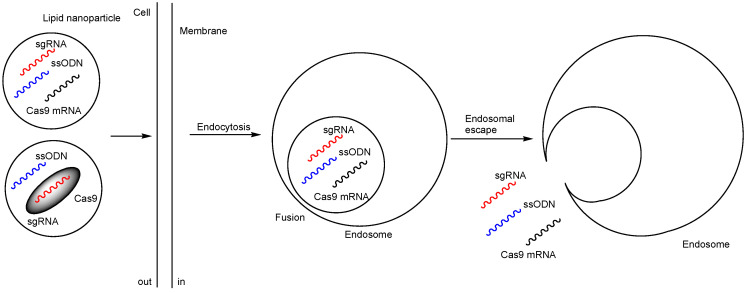
The outline of a lipid nanoparticle (LNP)-based Cas9 RNP delivery system uses optimally designed single-stranded oligonucleotides (ssODNs). The mechanism of endosomal escape is unknown; however, it is likely due to membrane fusion, although this remains uncertain.

**Figure 6:**
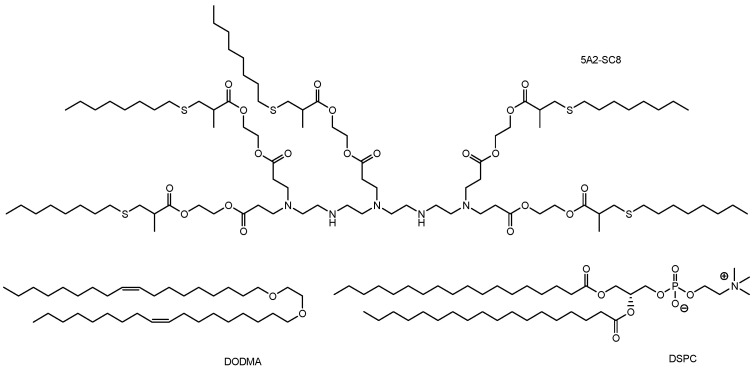
The structures of lipid nanoparticle components such as 5A2-SC8, 1,2-dioleyloxy-3-dimethylaminopropane (DODMA), and 1,2-distearoyl-sn-glycero-3-phosphocholine (DSPC).

**Figure 7:**
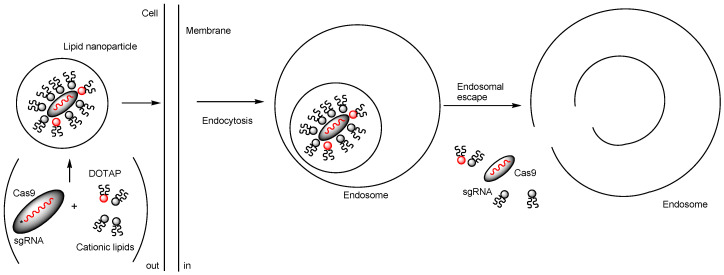
The pathway of LNPs encapsulating Cas9 ribonucleoprotein (Cas9 RNP), surrounded by cationic lipids such as 5A2-SC8 and 1,2-dioleoyl-3-trimethylammonium-propane (DOTAP) (depicted by a red circle), is described. Cas9 RNP is a complex of Cas9 protein and single-guide RNA (sgRNA). The LNPs are internalized into cells via lipid raft-mediated endocytosis. Although the mechanisms of endosomal escape remain unknown, DOTAP, as a permanent cationic lipid, may disrupt the membranes of both LNPs and endosomes.

**Figure 8:**
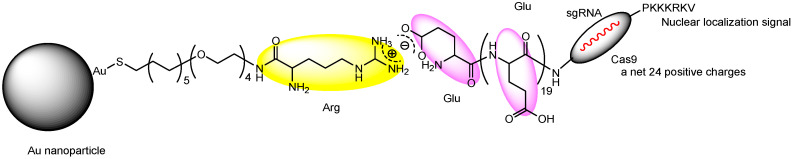
The structure of the nanoassembly is composed of Cas9 ribonucleoproteins (RNPs) with an oligo (20) Glu tag (Cas9E20) and a gold nanoparticle, linked through electrostatic connections between Glu (purple) and Arg (yellow).

Additionally, (ii) a lipid nanoparticle (LNP)-based Cas RNP delivery system incorporating optimally designed single-stranded oligonucleotides (ssODNs) was developed (Figure 5). The LNP formulation consisted of 1,10-[[2-[4-[2-[2-[bis(2-hydroxydodecyl)amino]ethylamino]ethyl]piperazin-1-yl]ethyl]azanediyl]bis(dodecan-2-ol) (C12-200), 1,2-dioleoyl-sn-glycero-3-phosphoethanolamine (DOPE), cholesterol, DMG-PEG, and 1,2-dioleoyl-3-trimethylammonium-propane (DOTAP) (Figure 4), with molar ratios of 35:16:46.5:2.5:0.25, respectively. Cas9 RNP-ssODN complexes remain stable at temperatures below 25 °C but release ssODNs in the cytosol at 37 °C under physiological conditions. Regarding gene editing efficiency, RNP-loaded LNPs containing anti-TTR sgRNA (sgTTR-G269) and ssODNs designed with a 50% complementation rate (calculated Tm: 30 °C) achieved the highest suppression of TTR protein levels in serum, reducing them by 34% one week after intravenous administration in mice. Similarly, RNP-loaded LNPs containing anti-TTR sgRNA (sgTTR-G211) and ssODNs designed with a 40% complementation rate (calculated Tm: 26 °C) achieved the highest suppression of TTR protein levels, reducing them by 56% [21]. It has been suggested that the affinity between ssODNs and RNPs plays a role in suppression activity. An LNP-based Cas RNP delivery system utilizing ssODNs may help avoid interactions with off-target sequences within cells. Typically, ssODNs, composed of no more than 200 nt, contain a ≥30-nt homology arm at both the 5′ and 3′ ends, which can pair with both the DNA donor of sgRNA and the target site [22]. The mechanism of endosomal escape in this case is unknown; however, it is likely due to membrane fusion, although this is not certain. It is known that multicomponent lipoplexes, incorporating three to six lipid species simultaneously, were fused to the endosomal membrane [23]. It was not believed that PEGs played a role in endosomal escape because DMG-PEG was present with a relatively low content (0.25%). Alternatively, ionic lipids might induce local membrane disruption [24]. From the perspective of absorption, distribution, metabolism, and excretion (ADME), both RNP-loaded LNPs and free RNP predominantly migrated to the liver after intravenous administration, although they were also observed in the spleen, kidney, and lungs to a lesser extent. RNP-loaded LNPs accumulated in the liver, as it is a key organ for metabolism within the reticuloendothelial system. It is also suggested that phagocytic cells, such as macrophages, engulf LNPs. PEGs on nanoparticles help evade macrophage uptake by reducing protein attachment. It has been reported that polyglycerol on nanoparticles prevents macrophage uptake more effectively than PEG [25]. In other cases of nanodelivery, it is well-known that nanoparticles are spontaneously accumulated in solid cancer tissues due to passive targeting, which is based on the enhanced permeability and retention (EPR) effect [26,27]. Nanoparticles pass through the leaky vessels of neovasculature into solid tumor parenchyma, where the lymphatic system is underdeveloped. Eventually, in this case, with respect to distribution, intravenously injected LNPs encapsulating Cas9-RNP (termed pLNP) and HDR template (pLNP-HDR) (245 ± 4 nm in diameter) via tail vein injections delivered Cas9-RNPs primarily to the liver, with additional presence in the spleen and lungs of Ai9 female mice. On the other hand, intravenously injected LNPs encapsulating Cas9 mRNA and sgRNA (termed mLNP) and HDR template (mLNP-HDR) (150 ± 2 nm in diameter) via tail vein injections delivered Cas9 mRNA mainly to the liver. mLNP resulted in 60% gene knockout in hepatocytes in mice. Both LNPs were composed of C12-200, DOPE, cholesterol, DMG-PEG, and DOTAP. It is likely that Cas9 mRNA was translated into Cas9 protein in situ, while Cas9-RNP was short-lived. In fact, mLNP-HDR surpassed pLNP-HDR in gene editing efficiency in in vitro tests using HEK293T or HEPA1-6 cells [21]. It is believed that the spontaneous delivery of nanoparticles to the liver via the reticuloendothelial system is a form of passive targeting in a broad sense. Nonetheless, nanoparticles were also observed in the spleen, kidney, and lung, in addition to the liver. Therefore, distribution based on more active targeting is expected to help avoid serious off-target side effects.

(iii) Ionizable cationic lipids with a pKa of approximately 6.4 bind to negatively charged RNAs at the pH used during mixing, thereby protecting the RNA. Tumor suppressor genes such as *P53*, *PTEN*, and *RB1* were targeted by RNPs. The 5A2-DOT type of LNP was composed of 5A2-SC8/DOPE/cholesterol/DMG-PEG/DOTAP in a molar ratio of 15:15:30:3:7 (Figure 6). As a component, 5A2-SC8 is a degradable dendrimer containing multiple ester groups, while DOTAP is a permanently cationic lipid. The notation 5A2-DOT-n refers to LNPs with n mole% DOTAP incorporation. Intravenous administration of 5A2-DOT-5 LNPs encapsulating Cas9/sgP53/sgPTEN/sgRB1 RNPs resulted in visible tumor formation on the liver in adult C57BL/6 mice. Similarly, intravenous administration of 5A2-DOT-50 LNPs encapsulating Cas9/sgEml/sgAlk RNPs induced Eml4-Alk rearrangements, leading to lung tumor formation in adult C57BL/6 mice (Figure 7) [28]. The formation of tumors in the liver and lungs following administration of LNPs encapsulating RNPs targeting tumor suppressor genes suggests a lack of tissue selectivity in these LNPs. The internalization mechanism was identified as lipid raft-mediated endocytosis, which could be inhibited by methyl-β-cyclodextrin (MβCD) or at 4 °C. Although the exact mechanisms of endosomal escape remain unclear, DOTAP, a permanent cationic lipid highlighted in the study, may locally disrupt the membranes of LNPs and endosomes. The presence of cationic lipid–anionic lipid ion pairs has been suggested to cause disruption of the endosomal membrane by inducing a non-bilayer structure [24]. It is also believed that the membrane of PEG-coated LNPs is composed primarily of DMG-PEG, which lacks anionic phosphate groups, along with DOPE, which contains anionic phosphate groups. Thus, anionic DOPE may interact with cationic DOTAP. It is well-established that PEGylation on the surface of nanoparticles acts as a stealth polymer, suppressing aggregation, opsonization, and phagocytosis, thereby extending circulation time in drug delivery systems [29]. In this system, lipid raft-mediated endocytosis is believed to occur due to interactions with cholesterol present in the LNP membranes. Thus, PEGs did not sterically hinder their binding to the cell surface. However, membrane fusion between LNPs coated with hydrophilic PEGs and the lipid bilayer of the plasma membrane is unlikely, although it might occur depending on modifications to the PEG ends, similar to cell–cell fusion [30]. Additionally, free PEGs are commonly known to relax the lipid bilayer structure of cell membranes, thereby promoting adhesion between membranes during cell–cell fusion. Nevertheless, the endosomal escape mechanism was attributed to local membrane disruption caused by DOTAP.

(iv) Nanoassemblies composed of Cas9 RNP with oligo (20) glutamic acid tags (Cas9E20) targeting *PTEN* and gold nanoparticles, such as Arg-AuNPs, were developed using a linker that enabled electrostatic interactions between glutamic acid and arginine residues (Figure 8). Following tail-vein injection into BALB/c mice, these nanoassemblies exhibited significantly higher distribution in macrophages compared to T cells and B cells in the liver and spleen. This nanoparticle selectivity between macrophages and immune cells like T cells may be attributed to the reticuloendothelial system, which plays a critical role in host defense against infectious agents. The average gold content in sorted CD11+ macrophages was higher in the spleen compared to the liver. Nanoassemblies (285 ± 23 nm in diameter) administered via tail-vein injection into mice achieved gene editing efficiencies of over 8% in liver macrophages and over 4% in spleen macrophages [31]. The internalization mechanism of Cas9E20 into cells was not receptor-mediated endocytosis but membrane fusion facilitated by nanoassemblies, whose nanoparticle membrane consisted of linoleic acid. The uptake of nanoassemblies was not detected through fluorescence microscopy analysis of cell fluorescence. Instead, during the process of membrane fusion, the nanoassemblies integrated into the membrane and released Cas9E20 into the cytosol. Cas9E20 subsequently entered the nucleus, guided by a nuclear localization signal (PKKKRKV) [32]. However, this process might also involve macrophage phagocytosis, given the observed cell selectivity between macrophages and T cells.

**Figure 9:**
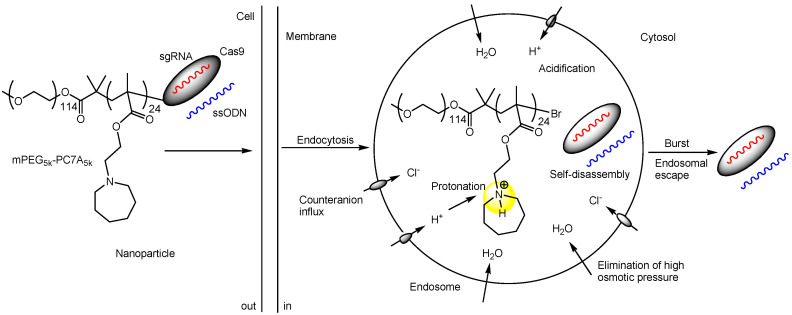
The pathway of nanoparticles, composed of the Cas9 ribonucleoprotein (RNP) formed by Cas9 and single-guide RNA (sgRNA), and the single-stranded oligonucleotide (ssODN) self-assembled with mPEG-PC7A through hydrophobic interactions, involves the release of the Cas9 RNP and ssODN complex into the cytosol via endocytosis. This process is followed by self-disassembly due to acidification in the endosomes and endosomal escape based on the proton sponge effect. As indicated in yellow, proton acceptance by amines in the endosomes triggers an influx of chloride ions and water, leading to osmotic rupture of the endosomal/lysosomal membrane.

**Figure 10:**
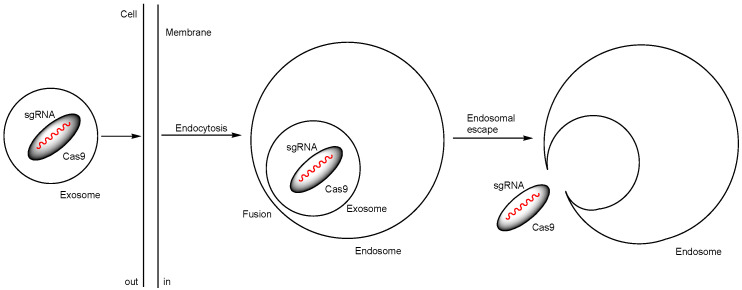
The pathway of exosome^RNP^ nanocomplex internalization involves endocytosis and endosomal escape of the Cas9 ribonucleoprotein (RNP), composed of Cas9 and single-guide RNA (sgRNA), with these components acting as their cargos via membrane fusion.

(v) Methoxy-poly(ethylene glycol)-b-poly [2-(azepan-1-yl)ethyl methacrylate] (mPEG-PC7A) contains ionizable PC7A units with both cationic and hydrophobic side groups. Consequently, Cas9 RNPs with ssODNs, facilitating genome editing via HDR, spontaneously form nanoparticles with mPEG-PC7A polymers due to hydrophobic interactions (termed HDR-NP) when the amino groups are not protonated. Conversely, they self-disassemble when the amino groups are protonated during endosomal acidification (Figure 9). Similarly, Cas9 RNPs facilitating genome editing via NHEJ spontaneously form nanoparticles with mPEG-PC7A polymers (termed NHEJ-NP). The Cas9 RNPs targeted a STOP cassette consisting of three SV40 polyA sequences to block transcription of the downstream tdTomato gene. NHEJ-NP (29.4 nm in diameter), administered intravenously, intratracheally, or intramuscularly, induced efficient gene editing in the liver, lungs, and skeletal muscle of mice, respectively. Intravenous administration of NHEJ-NP likely resulted in its distribution to the liver via the reticuloendothelial system. The site of administration plays a crucial role. Moreover, intramuscular injection of HDR-NP (33.3 nm in diameter) demonstrated muscle strength recovery in a Duchenne muscular dystrophy mouse model. Clathrin-dependent endocytosis (with an endosomal diameter of 85–150 nm) is the most prominent mode of endocytosis, although several mechanisms exist. The endocytosis of NHEJ-NPs in HEK293 cells was inhibited by chlorpromazine (an inhibitor of clathrin-dependent endocytosis), methyl-β-cyclodextrin, and genistein (both inhibitors of caveolin-dependent endocytosis). This indicates that clathrin-dependent and caveolin-dependent endocytosis occur simultaneously. The size of the nanoparticles is appropriate for cellular uptake through these pathways, whereas the Cas9 protein has a diameter of 7.5 nm, the sgRNA has a diameter of 5.5 nm. After endocytosis, HDR-NPs containing Cas9 RNPs with ssODNs as the payload and mPEG-PC7A polymers dissociated from the positively charged protonated polymers in endosomes and escaped into the cytosol via the proton sponge effect [33]. Interestingly, CPP-NHEJ-NPs, prepared using 50% CPP-PEG-PC7A and 50% mPEG-PC7A, were not internalized via endocytosis in HEK293 cells, unlike NHEJ-NPs. Their internalization was not inhibited by chlorpromazine or genistein. TAT (CYGRKKRRQRRR) was used as the CPP. Typically, positively charged TAT induces receptor-mediated endocytosis by utilizing negatively charged heparan sulfate proteoglycans (HSPGs) as receptors. It is unlikely that CPP-NHEJ-NPs (approximately 33.3 nm in diameter) cross the membrane via passive diffusion or direct translocation due to their overall molecular size, although both endocytosis and direct translocation are widely recognized as mechanisms for CPP internalization into cells. Thus, the internalization mechanisms of CPP-NHEJ-NPs remain uncertain. Possible mechanisms could include macropinocytosis, other less understood forms of endocytosis, or membrane disruption. While it is true that CPP-NHEJ-NPs were not internalized via endocytosis, the trajectories of fluorophore-labeled compounds often differ from those of their original counterparts due to alterations in physical properties. Furthermore, the in vivo distribution of HDR-NPs and CPP-NHEJ-NPs may vary after administration due to interactions between the positively charged TAT and endogenous materials, such as serum albumin.

(vi) Endosomal escape via the proton sponge effect was achieved using CPPs such as the Lys- and His-rich amphipathic peptide LAH5 (KKALLALALHHLAHLAHHLALALKKA). Nanocomplexes, measuring 200–400 nm in diameter and formed electrostatically by combining Cas9 RNP with cationic LAH5 peptides, were internalized via endocytosis, likely mediated by anionic HSPGs acting as receptors for receptor-mediated endocytosis. Upon acidification of the endosomes, the LAH5 peptides became protonated, causing an endosomal burst through the proton sponge effect and facilitating the release of the nanocomplexes into the cytosol. The nuclear localization signal (NLS) sequences on Cas9 directed it into the nucleus. Nanocomplexes targeting *CCR5* successfully achieved gene editing across the membrane in various cell lines [34]. Although this LAH5-based delivery strategy for CRISPR-Cas genetic engineering shows promise for in vitro and ex vivo applications, the current nanocomplex formulation is unlikely to be stable for in vivo applications. However, Cas9 RNP-loaded nanoparticles coated with LAH5 could be practical for in vivo use.

(vii) Exosomes are lipid bilayer membrane vesicles (40–160 nm in diameter) and a type of natural nanoparticle released by cells to facilitate cell-to-cell communication. It is well-established that cancer cells release exosomes containing miRNAs to prepare a favorable microenvironment for metastasis [35]. This discovery suggests that natural exosomes could be utilized for tissue-selective drug delivery. In fact, exosomes derived from hepatic stellate cells have demonstrated selectivity toward hepatic cells. Exosome^RNP^ nanocomplexes, created by loading Cas9 RNPs into purified exosomes isolated from hepatic stellate cells via electroporation, were successfully taken up by hepatocytes. Tail vein administration of Exosome^RNP^ targeting *PUMA* alleviated acute liver injury caused by a single overdose of acetaminophen. The p53 up-regulated modulator of apoptosis (PUMA) protein was significantly elevated in the livers of acetaminophen-treated mice. Similarly, tail vein administration of exosome^RNP^ targeting *CcnE1* alleviated chronic liver fibrosis caused by three intraperitoneal injections of CCl_4_ per week for five weeks. Cyclin E1 (CcnE1), a member of the cyclin-dependent protein kinase family, promotes the proliferation of hepatic stellate cells. Additionally, tail vein administration of exosome^RNP^ targeting *KAT5* improved orthotopic hepatocellular carcinoma (HCC) transplanted via hepatic portal injection. K (lysine) acetyltransferase 5 (KAT5) is essential for HCC growth. Studies revealed that exosomeRNP nanocomplexes were internalized via endocytosis, which was inhibited by chlorpromazine (a clathrin-dependent endocytosis inhibitor), genistein (a caveolin-dependent endocytosis inhibitor), or exposure to 4 °C [36]. The cargo delivery mechanisms of exosomes into cells are categorized into two modes: (a) fusion with the plasma membrane of recipient cells or (b) endosomal escape after endocytosis. The liver is a key metabolic organ. The exosomes taken up by hepatocytes may undergo degradation or follow the secretory pathway within the endo-lysosomal system, depending on the signal proteins on the surface of the exosomes or the endocytic internalization of bystander exosomes due to the high frequency of endocytosis in the liver. Nonetheless, it is believed that the endosomal escape mechanism of loaded Cas9 RNPs involves fusion between the endosome and the exosomes (Figure 10).

#### 2.2.2. Restricted Distribution to the Target Sites via Local Injections

Simple Cas9 RNP-loaded nanoparticles lack cell selectivity, leading to off-target side effects. To improve bioavailability and minimize such side effects, local injections can restrict distribution to specific target sites, compared to oral or intravenous administration, although intravenous administration is technically a form of injection. Local injections have been implemented in the eye, bone marrow cavity, and cranial regions. The blood–retinal barrier (BRB) and the blood–aqueous barrier (BAB) pose challenges for delivering drugs to the eyes. Therefore, local injections directly into the eye are effective. The cornea of the eye consists of three cell layers: the epithelial cell layer, the stromal layer, and the endothelial layer.

(viii) Intrastromal injection of LNP-encapsulated CRISPR targeting tdTomato in the loxP-3xStop-loxP-tdTomato reporter system resulted in successful transfection not only of stromal cells but also of endothelial cells in all wild-type eyes of 3-month-old hybrid B6129F1-loxP-3xStop-loxP-tdTomato mice. tdTomato is an exceptionally bright red fluorescent protein, capable of producing fluorescence detectable up to a depth of 1 cm from the epidermis [37]. Several Cas9 RNPs were tested for their ability to disrupt stop signals and activate tdTomato expression. Although the corneal stroma is separated from the single-cell-thick endothelium by a thin, acellular matrix known as Descemet’s membrane, the injected LNPs successfully crossed this barrier, as evidenced by tdTomato fluorescence. Moreover, Cas9 RNP-loaded LNPs corrected the Sey variant and restored FLAG-tagged PAX6 expression in 38% of ex vivo cortical neurons derived from a mouse model of aniridia, known as Small eye (Sey). This model carries the nonsense c.580G>T (p.G194X) variant in the *Pax6* gene [38]. The LNPs used in these experiments were composed of 1,2-dioleyloxy-3-dimethylaminopropane (DODMA), DOPE, 1,2-distearoyl-sn-glycero-3-phosphocholine (DSPC), and cholesterol (Figure 6). This delivery strategy, utilizing LNPs encapsulating RNP and DNA template mixtures via intrastromal injection, holds potential for CRISPR/Cas9-based genome editing therapies for corneal diseases.

**Figure 11:**
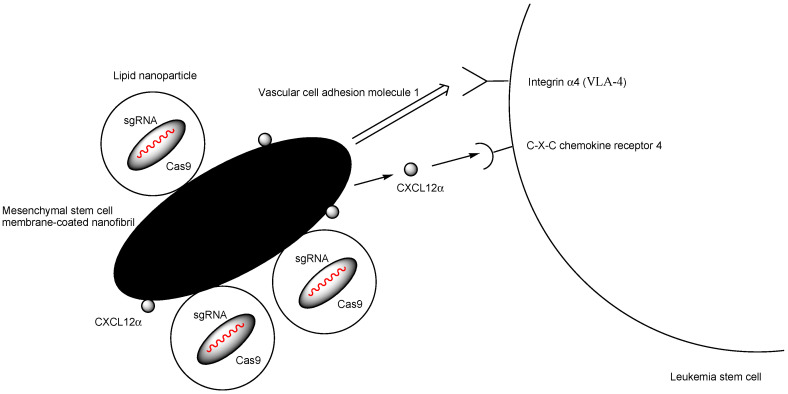
The interaction between leukemia stem cells and injected mesenchymal stem cell membrane-coated nanofibrils (MSCM-NFs) loaded with LNPs encapsulating Cas9 RNP targeting the critical genes interleukin-1 receptor accessory protein (IL1RAP) and CXCL12α was studied. CXCL12α, a ligand for C-X-C chemokine receptor 4 (CXCR4), recruited leukemia stem cells. As a result, vascular cell adhesion molecule 1 (VCAM-1) derived from the mesenchymal stem cell membrane bound integrin α4 (VLA-4) on the surface of leukemia stem cells. Endocytosed LNPs encapsulating Cas9 RNP induced efficient gene editing in leukemia stem cells following endosomal escape.

Leukemia stem cells (LSCs) sustain the progression of acute myeloid leukemia (AML), a type of cancer that originates in the blood-forming cells of the bone marrow, and contribute to disease relapse.

(ix) Mesenchymal stem cell membrane-coated nanofibrils (MSCM-NFs), loaded with (a) bioreducible LNPs encapsulating Cas9 RNP targeting the critical gene interleukin-1 receptor accessory protein (*IL1RAP*) in human LSCs and (b) CXCL12α, a ligand of the C-X-C chemokine receptor 4 (CXCR4) (Figure 11), were injected into the bone marrow cavity of mice. This approach successfully demonstrated that IL1RAP knockout reduced the colony-forming capacity of LSCs and decreased the leukemic burden [39]. CXCL12α facilitated the recruitment of leukemia cells. Consequently, vascular cell adhesion molecule 1 (VCAM-1), derived from the mesenchymal stem cell membrane (173.8 ± 46.4 nm in thickness) on nanofibrils (1.0 ± 0.2 μm in diameter and 22.0 ± 10.1 μm in length), bound to integrin α4 (VLA-4) on the surface of leukemia stem cells. LNPs (73.3–162.2 nm in diameter) encapsulating Cas9 RNPs, which separated from MSCM-NFs, were internalized into leukemia stem cells via endocytosis. They induced efficient gene editing after endosomal escape, likely through membrane fusion [40]. The endosomal diameter in clathrin-dependent endocytosis or clathrin- and caveolae-independent endocytosis is approximately 85–150 nm and around 90 nm, respectively, although the specific type of endocytosis that occurs remains uncertain. A single LNP could fit within an endosome. The SpCas9 protein has a molecular weight of 160 kDa and a hydrodynamic diameter of approximately 7.5 nm, while sgRNA has a molecular weight of around 31 kDa and a hydrodynamic diameter of about 5.5 nm [41]. Therefore, due to their size, several Cas9 RNP molecules could be encapsulated within one LNP. Since the target *IL1RAP* gene is located in the cell nucleus, efficient gene editing is successfully achieved.

The BBB significantly hinders drug delivery to the brain. Consequently, local injections into the brain serve as an alternative and urgent method for direct delivery. The potential of locally injecting Cas9 RNP into the brain was evaluated to assess genome editing using the tdTomato reporter system, similar to the intrastromal injections into the cornea described earlier. Although the tdTomato locus is relatively large, the expression of the tdTomato protein in a mouse model indicates the actual genome editing efficiency and the distribution of Cas9 RNP. Four repeats of the positively charged Simian Vacuolating Virus 40 nuclear localization sequences (SV40-NLS) were fused to the *N*-terminus, along with two repeats fused to the *C*-terminus of Cas9, enabling it to spontaneously move to the cell nucleus. In total, six SV40-NLS sequences were fused to Cas9 (4x-Cas9-2x). Cell-penetrant Cas9 RNPs were injected into the striatum of lox-stop-lox Ai9 mice. As a result, these cell-penetrant Cas9 RNPs edited more neurons in the region near the injection site, as indicated by tdTomato fluorescence. In contrast, transient RNP complexes delivered using AAV serotype 9 (Cas9-AAV) exhibited better diffusion throughout the brain. However, brains treated with Cas9-AAV showed significantly elevated *Cd3e* gene expression, indicative of an adaptive immune response. Meanwhile, brains treated with Cas9 RNPs experienced acute microglial activation, which could be mitigated by reducing endotoxin levels. Consequently, Cas9 RNPs demonstrated superior safety compared to Cas9-AAV [42]. The restricted distribution of Cas9 RNPs, along with their safety profile, could potentially be enhanced through the use of nanoparticles.

(x) Non-viral, biodegradable PEGylated nanocapsules (NCs) encapsulating Cas9 RNPs (less than 35 nm in diameter) were evaluated for genome editing in neurons following intracerebral injection. The optimal formulation utilized a molar ratio of acrylic acid (AA)/*N*-(3-aminopropyl)methacrylamide hydrochloride (APMA)/1-vinylimidazole (VI)/*N*,*N*′-bis(acryloyl)cystamine (BACA)/acrylate-polyethylene glycol (Ac-PEG)/RNP at 927/927/244/231/64/1. The small size of the NCs may facilitate their diffusion within the brain. Indeed, NCs loaded with RNPs containing anti-Ai14 sgRNA, administered via intracerebral injection into the healthy mouse striatum, demonstrated successful genome editing of striatal neurons as determined by the tdTomato reporter system. The Cas9 RNP targeting sites were located within each of the three SV40 polyA sequences in the STOP cassette. The removal of two SV40 polyA cassettes led to the expression of tdTomato. Genome editing was predominantly observed in medium spiny neurons (>80%), with occasional editing in cholinergic, calretinin, and parvalbumin interneurons [43]. The conjugation of neuron-specific ligands, such as the rabies virus glycoprotein-derived peptide (RVG peptide: YTIWMPENPRPGTPCDIFTNSRGKRASNG), a ligand for the α-7 subunit of nicotinic acetylcholine receptors (AchRs) in neuronal cells [44], could enhance specificity for neuron-targeted delivery. Moreover, conjugation of cell-penetrating peptides (CPPs) such as TAT may enhance cellular uptake. However, contrary to expectations, the addition of either CPP or RVG to the nanocapsules (NCs) did not significantly affect the neuronal editing efficiency or the size of the edited brain area in this assay system. The Cas9 RNP-loaded NCs facilitated endosomal escape via imidazole-containing monomers (VIs) and amine-containing monomers (APMAs), likely due to the proton sponge effect. Subsequently, the Cas9 RNPs were separated from the NCs through cleavage of the glutathione-responsive cross-linker in the cytosol.

(xi) CRISPR–gold targeting the metabotropic glutamate receptor 5 (*mGluR5*) gene efficiently reduced local mGluR5 protein levels in the striatum by 40–50% following intracranial injection into the brains of *Fmr1* knockout mice, a model of fragile X syndrome (FXS). This reduction in mGluR5 protein levels ultimately led to a decrease in hyperlocomotor activities, such as excessive digging and jumping behavior, in the Fmr1 knockout mice [45]. FXS [46] is an inherited genetic disorder caused by mutations in the fragile X mental retardation 1 (*FMR1*) gene. The FMR1 gene contains a CGG repeat in the 5′-untranslated region. Silencing the FMR1 gene prevents the expression of the FMR protein (FMRP). Low-molecular-weight metabotropic glutamate receptor 5 (mGluR5) antagonists have been developed for the treatment of FXS [47]. mGluR5 is an excitatory G-protein-coupled receptor. According to the mGluR theory of FXS, glutamatergic signaling through mGluR5 is enhanced in the absence of FMRP, thereby inducing various neurological and neuropsychiatric features of FXS. At present, low-molecular-weight mGluR5 antagonists are unlikely to be clinically available for the treatment of FXS. Local injections into the brain, which bypass the BBB, may be a significant strategy for treating inherited genetic diseases, including FXS.

(xii) The development and progression of psoriasis are associated with NLRP3 inflammasomes, which are large protein complexes that play a crucial role in regulating the innate immune response and inflammation. Therefore, genome editing of the NLRP3 inflammasome could alleviate the inflammatory symptoms of psoriasis. Polyamidoamine nanocomplexes (named GBLA-22), coated with phenylboronate and lipoic acid moieties as ligands and containing Cas9 RNP targeting *NLRP3*, achieved a 33.7% gene disruption at the NLRP3 locus in psoriatic skin tissues in an in vivo assay. This was accomplished via subcutaneous injection into the psoriatic skin on the back through two-point injections at the upper and lower parts of the area, using an imiquimod-induced psoriatic mouse model. The treatment led to a reduction in the levels of IL-1β, IL-17, IL-18, TNF-α, and IL-12/23p40 [48]. Psoriatic model mice were generated by applying imiquimod cream daily to a shaved 2 cm × 2 cm area of skin for seven consecutive days. Phenylboronate and lipoic acid moieties were identified as crucial ligands for protein binding, endocytosis, endosomal escape, and intracellular protein release. The cellular uptake mechanism was determined to be lipid raft-mediated endocytosis, as it was inhibited by MβCD (a cholesterol-depleting reagent) or at 4 °C but remained unaffected by cytochalasin D (a macropinocytosis inhibitor), genistein (a caveolae-dependent endocytosis inhibitor), or chlorpromazine (a clathrin-dependent endocytosis inhibitor). Furthermore, the endosomal escape mechanism was suggested to occur via membrane disruption. Following subcutaneous injection, GBLA-22-Cy5/RNP complexes were detected in the skin and liver from days 1 to 5, as indicated by Cy5 fluorescence, suggesting that these organs serve as the primary sites of nanoparticle metabolism.

(xiii) Overexpressed microRNA-21 (miR-21) in human hepatocellular carcinoma inhibits the expression of tumor suppressor genes. Therefore, miR-21-sensitive nanodelivery systems can selectively induce apoptosis in cancer cells. Nanocapsules composed of nanoassembled, engineered DNAzyme shells encasing Cas9 RNP targeting the *MIR-21* gene—referred to as miR-21-responsive DNAzyme-functionalized nanocapsules (R-DN)—demonstrated significant tumor growth inhibition of up to 75.94% in an in vivo assay. This was achieved through intratumoral injection in a HepG2 tumor mouse model, which was established by subcutaneous armpit injection of 2 × 10^6^ cells in a PBS/Matrigel (1:1, *v*/*v*) mixture [49]. In general, DNAzyme is a catalytic single-stranded DNA that binds to and cleaves target RNA in a sequence-specific manner. R-DN entered cancer cells via endocytosis, which was inhibited by MβCD (a cholesterol-depleting reagent) and chlorpromazine (a clathrin-dependent endocytosis inhibitor). After endosomal escape—through mechanisms that remain unclear—cytosolic miR-21 was believed to interact with R-DN. It was discovered that as little as 1.7 pM of miR-21 could trigger the on-demand release of Cas9 RNP from R-DN through a conformational change in the DNAzyme upon miR-21 binding. The released Cas9 RNP then edited the miR-21 gene, leading to the upregulation of tumor suppressor genes that induce apoptosis. This process established a positive feedback-driven autonomous catabolic cycle between miR-21 and R-DN.

Nonetheless, local injections cause physical pain to patients, although Cas9 RNPs are reliably delivered to the target sites and remain in the restricted area, such as the cranial cavity. Therefore, non-invasive administration methods should be developed.

#### 2.2.3. Selective and Effective Distribution Through Non-Invasive Methods

Selective and effective distribution through non-invasive methods, combining active targeting with a deliberate approach and passive targeting with a laissez-faire approach, is anticipated to minimize off-target side effects. Nevertheless, differences between target sites and other healthy regions persist due to inherent biological mechanisms rooted in structural principles. Cell surfaces are generally covered with negatively charged HSPGs, which interact with CPPs to facilitate receptor-mediated endocytosis. However, the surface of cancer cells exhibits a more negatively charged characteristic due to their unique metabolic processes, which produce lactate anions as part of the Warburg effect [50]. The secretion of lactate anions displaces cations such as H^+^ and Na^+^ from the cancer cell surface, enhancing the negative charge. The Warburg effect also creates a mildly acidic extracellular environment (pH 6.6–6.8) in cancer tissues [51]. Furthermore, phospholipids such as phosphatidylethanolamine and phosphatidylserine flip to the outer surface of cancer cells [52]. Nonetheless, more pronounced differences may be necessary for effective targeting. The potential for achieving cell selectivity between cancer and normal cells remains an area for discovery. Notably, RGD peptides are well-known for inducing receptor-mediated endocytosis by utilizing αvβ_3_ integrins as receptors on the cancer cell surface [53]. Cell-selective internalization was achieved in cancer drug delivery systems using iRGD (c(CRGDKGPDC)) as a tumor-homing ligand.

(xiv) Nanoparticles coated with iRGD were used for the co-delivery of Cas9 RNP targeting the *Nrf2* gene and the antitumor photosensitizer chlorin e6 (Ce6). These nanoparticles, with diameters ranging from 84 to 92 nm, were administered intravenously and internalized into cells via receptor-mediated endocytosis in a mouse tumor model. In this model, cellosaurus CNE-2 cells, derived from nasopharyngeal carcinoma, were subcutaneously injected into the mice’s armpits. Near-infrared irradiation, administered 12 h after nanoparticle delivery, induced the generation of reactive oxygen species (ROS) by Ce6. These ROS destabilized lysosomal membranes, enabling the nanoparticles to escape the lysosome. This facilitated genome editing, thereby increasing tumor sensitivity to ROS [54]. The ROS generated by Ce6 exhibited a synergistic antitumor effect, inducing apoptosis in addition to destabilizing lysosomal membranes. Furthermore, the Nrf2 protein is implicated in promoting angiogenesis. Hypoxia-inducible factor 1α (HIF1α) and vascular endothelial growth factor-A (VEGF-A) are key angiogenic factors. Treatment with Ce6 and Cas9 RNP-encapsulated nanoparticles led to a reduction in HIF1α and VEGF-A levels by 50.0% and 44.6%, respectively, compared to levels observed with Ce6-encapsulated nanoparticles without Cas9 RNP. Consequently, intravenously administered nanoparticles encapsulating both Ce6 and Cas9 RNP demonstrated a more significant antitumor effect than those lacking iRGD.

(xv) Intravenously administered glutathione-responsive silica nanocapsules (SNCs) conjugated with glucose and an RVG peptide, encapsulating Cas9 mRNA along with either App^659^ sgRNA or Th sgRNA, achieved up to 6.1% editing efficiency of the amyloid precursor protein (*App*) gene in the thalamus/hypothalamus (resulting in a 19.1% reduction in intact APP expression levels) and up to 3.9% editing efficiency of the tyrosine hydroxylase (*Th*) gene in the thalamus/hypothalamus (resulting in a 30.3% reduction in Th expression levels), respectively, in C57BL/6J wild-type mice. These SNCs crossed the BBB via receptor-mediated transcytosis, utilizing glucose transporter-1 (GLUT1) and the α-7 AchR of endothelial cells. Subsequently, the SNCs were internalized into neurons via receptor-mediated endocytosis, utilizing the α-7 AchR of neuronal cells. The GSH-responsive SNCs released their cargo into the cytosol following endo/lysosomal escape, likely facilitated by the proton sponge effect of the imidazole-containing chains [55]. Overall, the strategy of using GSH-responsive, imidazole-rich SNCs conjugated with glucose and the RVG peptide was highly effective in achieving targeted delivery. However, the precise mechanisms underlying endosomal escape remain unclear.

(xvi) Intravenously injected, thin disulfide-cross-linked polymeric shell nanocapsules decorated with angiopep-2 peptide and encapsulating Cas9 RNP (approximately 30 nm in diameter) achieved high *PLK1* gene editing efficiency in brain tumors, reaching up to 38.1% in orthotopic CSC2-Luc GSC tumor-bearing mice [56]. The angiopep-2 peptide (TFFYGGSRGKRNNFKTEEY) is a ligand that binds to low-density lipoprotein receptor-related protein-1 (LRP-1), which is highly expressed on both BBB endothelial cells and glioblastoma (GBM) cells. These nanocapsules crossed the BBB via receptor-mediated transcytosis using LRP-1 as a receptor, as well as through passive diffusion induced by BBB disruption caused by GBM. Subsequently, the nanocapsules were internalized into the neurons via receptor-mediated endocytosis, utilizing LRP-1.

Similarly, (xvii) a brain-targeted CRISPR/Cas9-based nanomedicine was developed by fabricating angiopep-2-decorated, guanidinium- and fluorine-functionalized polymeric nanoparticles that loaded Cas9/gRNA RNP for the treatment of GBM. These nanoparticles exhibited approximately 32% gene knockout and a 67% reduction in the protein levels of the targeted proto-oncogene, polo-like kinase 1 (PLK1), in in vitro tests using U87MG cells. This gene knockout was sufficient to prolong the median survival time of mice bearing orthotopic glioblastoma to 40 days, without serious side effects or off-target effects [57].

**Table 1:** Summary of the Cas9 ribonucleoprotein (RNP)-encapsulated nanoparticles discussed in this review.

#	Formulations	Administration	Diseases	Targeting Gene	Results	Status	Refs.
i	NTLA-2001 (LNPs encapsulating Cas9 mRNA and sgRNA targeting TTR	Intravenous injection	TTR amyloidosis	*TTR*	Preclinical studies showed that at day 28 the mean reduction in serum TTR protein concentration was 52% in the group that received a dose of 0.1 mg per kilogram and was 87% in the group that received a dose of 0.3 mg per kilogram.	Phase 1 clinical trial (NCT04601051)	[17]
ii	LNP-based Cas RNP delivery system using optimally designed ssODNs	Intravenous injection	TTR amyloidosis	*TTR*	RNP-loaded LNPs with anti-TTR sgRNA (sgTTR-G269) and designed ssODNs with a complementation rate of 50% (calculated Tm: 30 °C) suppressed TTR the highest (34%) as TTR protein levels in serum, quantified 1 week after intravenous administration in mice.	Basic research	[21]
iii	5A2-DOT-5 LNPs encapsulating Cas9/sgP53/sgPTEN/sgRB1 RNPs	Intravenous injection	Induction of cancer	*P53*, *PTEN*, or *RB1*	Generation of visible tumors on the liver in adult C57BL/6 mice.	Basic research	[28]
iv	Nanoassemblies composed of Cas9 RNP with oligo (20) Glu tags (Cas9E20) and Arg-AuNPs electrostatically connected between Glu and Arg	Intravenous injection	-	*PTEN*	>8% and >4% gene editing efficiency in macrophages of the liver and the spleen, respectively.	Basic research	[31]
v	Nanoparticles composed of Cas9 RNPs with ssODNs and mPEG-PC7A	Intravenous, intratracheal, or intramuscular injection	-	A STOP cassette that consists of three SV40 polyA sequences to prevent transcription of the downstream tdTomato	Intravenously, intratracheally, and intramuscularly injected NHEJ-NP (29.4 nm in diameter) induced efficient gene editing in mouse liver, lung, and skeletal muscle, respectively.	Basic research	[33]
vi	Nanocomplexes (200–400 nm in diameter) electrostatically composed of a Cas9 RNP and cationic LAH5 peptides	-	-	*CCR5*	Nanocomplexes targeting CCR5 exhibited the gene editing across the membrane in diverse cell lines.	Basic research	[34]
vii	Exosome^RNP^ nanocomplexes, prepared by loading Cas9 RNPs into purified exosomes isolated from hepatic stellate cells through electroporation	Intravenous injection	liver injury, chronic liver fibrosis, or hepatocellular carcinoma	*PUMA*, *CcnE1*, or *KAT5*	Exosome^RNP^ targeting *PUMA* ameliorated acute liver injury. Exosome^RNP^ targeting *CcnE1* ameliorated chronic liver fibrosis. Exosome^RNP^ targeting *KAT5* administered by tail vein injections ameliorated orthotopic hepatocellular carcinoma (HCC).	Basic research	[36]
viii	LNP-encapsulated tdTomato-targeted CRISPR strategy in the loxP-3xStop-loxP-tdTomato reporter system	Intrastromal injection	corneal diseases	A STOP cassette that consists of three SV40 polyA sequences to prevent transcription of the downstream tdTomato	Transfection not only of the stromal cells but also of the endothelial cells in all wild-type eyes of 3-month-old hybrid B6129F1-loxP-3xStop-loxP-tdTomato wild-type mice.	Basic research	[37]
ix	MSCM-NFs loading (a) bioreducible LNPs encapsulating Cas9 RNP targeting the critical gene IL1RAP in human LSCs and (b) CXCL12α that is a ligand of CXCR4	Injection into the bone marrow cavity	acute myeloid leukemia	*IL1RAP* or *CXCL12α*	*IL1RAP* knockout reduced LSC colony-forming capacity and leukemic burden.	Basic research	[39]
x	PEGylated nanocapsules encapsulating Cas9 RNPs	Intracerebral injection	CNS diseases	A STOP cassette that consists of three SV40 polyA sequences to prevent transcription of the downstream tdTomato	Genome editing of striatal neurons, which was determined by the tdTomato reporter system.	Basic research	[43]
xi	CRISPR–gold targeting the mGluR5 gene	Intracranial injection	fragile X syndrome	*mGluR5*	Reduction in local mGluR5 protein levels in the striatum by 40–50% after an intracranial injection into the brains of Fmr1 knockout mice and eventual reduction in hyperlocomotor activities such as excessive digging and jumping behavior.	Basic research	[45]
xii	Nanocomplexes coated with phenylboronate and lipoic acid and containing Cas9 RNP	Subcutaneous injection	psoriasis	*NLRP3*	33.7% gene disruption at the NLRP3 locus in psoriatic skin tissues.	Basic research	[48]
xiii	Nanocapsules composed of nanoassembled, engineered DNAzyme shells encasing Cas9 RNP	Intratumoral injection	cancer	*MIR-21*	Tumor growth inhibition of up to 75.94%.	Basic research	[49]
xiv	Nanoparticles encapsulating Cas9 RNP targeting *Nrf2* gene and antitumor photosensitizer chlorin e6 (Ce6) and covered with iRGD	Intravenous injection	cancer	*Nrf2*	The Nrf2 protein is associated with angiogenesis promotion. Hypoxia-inducible factor 1α (HIF1α) and vascular endothelial growth factor-A (VEGF-A) are representative angiogenetic factors. Ce6 and Cas9 RNP-encapsulated NP treatment resulted in 50.0 and 44.6% reduction in HIF1α and VEGF-A levels, respectively.	Basic research	[54]
xv	GSH-responsive SNCs conjugated with glucose and RVG peptide and encapsulating Cas9 mRNA and App659 sgRNA or Th sgRNA	Intravenous injection	CNS diseases including Alzheimer’s disease	*App659* or *Th*	Up to 6.1% amyloid precursor protein (*App*) gene editing efficiency for thalamus/hypothalamus (resulting in 19.1% reduction in the expression level of intact APP) or up to 3.9% tyrosine hydroxylase (*Th*) gene editing efficiency for thalamus/hypothalamus (resulting in 30.3% reduction in the expression level of Th), respectively.	Basic research	[55]
xvi	Disulfide-cross-linked polymeric shell nanocapsules decorated with angiopep-2 peptide, encapsulating Cas9 RNP (approx. 30 nm in diameter)	Intravenous injection	glioblastoma	*PLK1*	High PLK1 gene editing efficiency in a brain tumor (up to 38.1%) in orthotopic CSC2-Luc GSC tumor-bearing mice.	Basic research	[56]
xvii	CRISPR/Cas9-based nanomedicine by fabricating an angiopep-2 decorated, guanidinium and fluorine functionalized polymeric NPs loading Cas9/gRNA RNP	-	glioblastoma	*PLK1*	32% gene knockout and 67% protein reduction in the proto-oncogene polo-like kinase 1 (PLK1) (in vitro).	Basic research	[57]
xviii	LNPs and polymer Pluronic F_127_-encapsulated CRISPR/Cas plasmid	Oral administration	colitis-associated colorectal cancer	*CD98*	Reduction in CD98 expression and demonstration of therapeutic efficacy against CAC.	Basic research	[58]
xix	Cas9 RNP-encapsulated NPs covered with anti-TfR and anti-α-7 AchR bispecific mAbs	Intravenous injection	CNS diseases	Certain proteins involved in CNS diseases	Under analysis in Tashima lab.	Basic research	-

Abbreviations. refs; references, LNP; lipid nanoparticle, mRNA; messenger ribonucleic acid, TTR; transthyretin, ssODNs; single stranded oligonucleotides, MSCM-NFs; mesenchymal stem cell membrane–coated nanofibrils, IL1RAP; interleukin-1 receptor accessory protein, CXCR4; C-X-C chemokine receptor 4, CNS; central nervous system, mGluR5; metabotropic glutamate receptor 5, SNC; silica nanocapsule, Th; tyrosine hydroxylase.

(xviii) Anti-colon disease drugs can be administered orally if they are resistant to degradation by stomach acid or proteases and are not absorbed in the small intestine. The progression of ulcerative colitis (UC) and colitis-associated colorectal cancer (CAC) is linked to the overexpression of CD98. P_127_M@pCD98s, composed of negatively charged LNPs derived from mulberry leaves and the U.S. Food and Drug Administration (FDA)-approved polymer Pluronic F_127_ (P127), encapsulate a CRISPR/Cas plasmid targeting *CD98* (pCD98) with an approximate diameter of 267.2 nm. In an in vivo assay using a CAC mouse model, oral administration of P127M@pCD98s significantly reduced CD98 expression and demonstrated therapeutic efficacy against CAC [58]. The CRISPR/Cas plasmid encodes both sgRNA and the Cas9 protein. P127M@pCD98s penetrated the mucus layer, accumulated in inflamed colon tissues, and were internalized by colon epithelial cells and macrophages via galactose receptor-mediated endocytosis, facilitated by the galactose end groups. After endosomal/lysosomal escape, pCD98 was released and became active. Macrophages are known to express CD98. However, since LNPs with a diameter of 267.2 nm are relatively large, they may not fit into endosomes. Therefore, LNPs might undergo size reduction during transport to facilitate internalization via galactose receptor-mediated endocytosis in colon epithelial cells and macrophages. Alternatively, the tight junctions between colon epithelial cells in CAC might be partially disrupted, enabling passive diffusion of LNPs. Subsequently, LNPs might enter macrophages via macropinocytosis, likely occurring within the reticuloendothelial system.

Therefore, a selective and effective distribution of Cas9 RNP-encapsulated nanoparticles in a non-invasive manner has been developed.

#### 2.2.4. Promising Delivery of Intravenously Administered Cas9 RNP-Encapsulated Nanoparticles to the Brain

It is well established that clustering induces endocytosis [59,60,61]. Therefore, a multiligand strategy on nanoparticles is more likely to promote cluster formation. For instance, SNCs conjugated with glucose as a GLUT1 ligand and RVG peptide as an α-7 AchR ligand were designed using a multiligand approach to target capillary endothelial cells [43]. The expression of receptors involved in endocytosis is often not limited to target cells. For example, HSPGs, which serve as receptors for CPPs like TAT, are expressed ubiquitously. A single-ligand strategy may result in off-target side effects. In contrast, a multiligand strategy is more likely to stochastically bind to specific regions that act as entry points to target sites, especially within systemic circulation.

The ligand–receptor selectivity of monoclonal antibodies is higher than that of CPPs or THPs. Consequently, Cas9 RNP-encapsulated nanoparticles coated with monoclonal antibodies can bind tightly and selectively to receptors that facilitate endocytosis or transcytosis. Notably, the clinically approved J-Brain Cargo^®^ system [62] enables antibody–drug conjugates to cross the BBB through receptor-mediated transcytosis via the transferrin receptor in capillary endothelial cells. Thus, Cas9 RNP-encapsulated nanoparticles coated with monoclonal antibodies targeting the transferrin receptor can cross the BBB via receptor-mediated transcytosis. Additionally, nanoparticles coated with bispecific monoclonal antibodies targeting both the transferrin receptor and the α-7 AchR can subsequently enter neurons after transendothelial migration.

## 3. Conclusions

The CRISPR/Cas9 gene editing technique is a powerful tool for precisely cutting and modifying DNA. However, delivering Cas9 RNPs within living organisms presents challenges, including low cell selectivity and bioavailability. While internalization into cells such as fertilized eggs, CAR-T cells, or MSCs can be achieved using methods like lipofection (cationic lipid-mediated transfection), electroporation (electric pulse methods), viral vector-based gene delivery, or microinjection, these approaches have limitations. Utilizing carrier nanoparticles coated with vectors such as monoclonal antibodies, CPPs, or THPs offers a promising solution to these challenges [Table 1]. Nanoparticles protect Cas9 RNPs from enzymatic degradation, while the vectors on nanoparticles facilitate receptor-mediated endocytosis or transcytosis by targeting tissue-specific receptors. Endosomal escape, enabling the release of Cas9 RNPs, is achieved through mechanisms such as membrane disruption via the proton sponge effect [33], ionic lipid-mediated local membrane disruption [24], fusion between endosomes and LNPs [23], or other strategies. Since Cas9 RNPs cannot cross membranes via passive diffusion, carefully designed Cas9 RNP-encapsulated nanoparticles coated with appropriate vectors can effectively enter target cells through receptor-mediated endocytosis, followed by successful gene editing after endosomal escape, leveraging the biological machinery system. Intravenously administered Cas9 RNP-encapsulated nanoparticles, coated with suitable vectors such as glucose and an RVG peptide, successfully crossed the BBB via receptor-mediated transcytosis utilizing GLUT1 and α-7 AchR in capillary endothelial cells, respectively. These nanoparticles were subsequently internalized into cells, including neurons, through receptor-mediated endocytosis via α-7 AchR. Ultimately, they facilitated gene editing for central nervous system (CNS) diseases following endosomal escape [43]. Conversely, intravenously administered Cas9 RNP-encapsulated nanoparticles, coated with suitable vectors such as iRGD, entered cancer cells via receptor-mediated endocytosis mediated by αvβ3 integrins and ultimately facilitated gene editing following endosomal escape [54].

Intelligent nanoparticles coated with vectors, such as specific membrane receptor ligands that induce endocytosis/transcytosis, could address scalability, reproducibility, and safety concerns in clinical translation. Notably, NTLA-2001 (NCT04601051) is an intravenously administered lipid nanoparticle formulation encapsulating Cas9 mRNA and sgRNA targeting *TTR*. NTLA-2001 forms complexes with APOE, enabling receptor-mediated endocytosis via LDLR in the liver, ultimately leading to reduced serum TTR protein expression through *TTR* gene knockout (Figure 3) [17]. Cas9 RNP-encapsulated nanoparticles coated with appropriate vectors are expected to be clinically introduced soon. However, human germline editing remains a topic of debate, as such modifications are inherited by future generations [63]. Therefore, the use of Cas9 RNP-encapsulated nanoparticles should be strictly limited to disease treatment. Additionally, Casgevy^®^ was clinically approved by the FDA in December 2023 for the treatment of sickle cell disease. This therapy involves using modified hematopoietic (blood) stem cells taken from the patient’s bone marrow. It is the first FDA-approved treatment utilizing CRISPR/Cas9, although it does not rely on a nanodelivery system [64].

Regarding scalability [65], large-scale production must be established. Developing scalable methods for nanoparticle production with high reproducibility and consistency is essential for translating these technologies into clinical and industrial applications [66,67,68,69,70]. This scalability issue can be addressed by focusing on (a) establishing robust production processes that ensure nanoparticles are consistently produced at a larger scale, (b) optimizing nanoparticle synthesis to maintain high-quality standards while increasing production capacity, (c) implementing standardization and quality control measures at each stage of production, (d) automating and integrating manufacturing steps to maintain reproducibility and consistency, (e) ensuring regulatory compliance, which is crucial for successful translation into clinical and industrial applications, and (f) achieving cost efficiency for large-scale commercialization.

Regarding reproducibility [66,67,68,69,70], efficient and stable encapsulation methods need to be developed. Achieving effective encapsulation of RNPs while maintaining their stability remains a significant challenge. Ensuring the structural and functional integrity of RNPs during formulation and storage is crucial for their effectiveness. The optimization of encapsulation techniques is based on (a) the selection of suitable carriers, such as LNPs, and (b) encapsulation methods.

Regarding safety [71], toxicity and biocompatibility are important considerations. Certain materials used for nanoparticle functionalization, such as ligands, may induce toxicity or trigger adverse immune responses, thereby limiting their clinical applicability. (a) Utilizing highly selective ligands, such as monoclonal antibodies, CPPs, or THPs, can help prevent off-target effects caused by incorrect distribution. Additionally, (b) PEG modification on the surface of nanoparticles is known to reduce immune responses.

Nonetheless, the CRISPR/Cas9 system has the potential to create new biological machinery through precise molecular design. Medicinal chemists and pharmaceutical scientists are expected to develop innovative drugs based on Cas9 RNP-encapsulated nanoparticles to treat patients suffering from genetic disorders.

## Data Availability

Data available in a publicly accessible repository. The data presented in this study are openly available in References below. ClinicalTrials.gov Identifier can be found at https://clinicaltrials.gov/ (accessed on 1 January 2025).
